# *SALL4* Gene Expression in Acute Myeloid Leukemia

**DOI:** 10.31557/APJCP.2019.20.10.3121

**Published:** 2019

**Authors:** Fatma Mahmoud Ibraheem, Ragia Badawy, Mahmoud Aly Ayoub, Naglaa Mostafa Hassan, Marwa Nabil Mostafa

**Affiliations:** 1 *Department of Clinical Pathology,*; 2 *Department of Medical Oncology, National Cancer Institute, Cairo University, Cario, Egypt. *

**Keywords:** *SALL4* gene, acute myeloid leukemia, National Cancer Institute, quantitative real-time PCR

## Abstract

**Objectives:**

*SALL4* gene was aberrantly expressed in many leukemia cell lines and primary leukemia cells of acute myeloid leukemia (AML) and precursor B-cell lymphoblastic leukemia/lymphomas. Its expression may be a useful marker to predict the diagnosis and the risk stratification of patients with AML.

**Methods:**

This study aimed to characterize the expression pattern of *SALL4* gene in adult patients with acute myeloid leukemia. Quantitative Real-time PCR was used to determine the expression level of the gene in peripheral blood of 52 Egyptian adult AML patients and 10 healthy control cases. Our study was done in the National Cancer Institute during the period of time between December 2014 and June 2015.

**Results:**

The observed data revealed that none of the studied controls expressed *SALL4* > 1.0 RQ and there was a highly statistically significant difference between cases and controls regarding *SALL4* gene expression where all cases showed higher expression of *SALL4* than controls with p value <0.001.

**Conclusion:**

In AML, *SALL4* is one of few genes that bridge the self-renewal properties of ESCs, normal HSCs and LSCs. Their expression is easily determined by real time PCR. They may be useful markers to predict prognosis and help to stratify patients into risk adapted groups. Further studies including increasing patient numbers are essential to understand the relations between *SALL4* gene expression and its prognostic impact.

## Introduction

Acute myeloid leukemia (AML) is a clonal disorder arises from the differentiation arrest of myeloid precursor and malignant proliferation of a bone marrow derived, self-renewing stem or progenitor cells in the bone marrow (BM) and blood. It can be classified into different subtypes based on the cell type and the degree of maturity according to WHO classification of hematological diseases (Jeonget al., 2011).

Over the past few years, remarkable progress has been made in identifying stem cell factors that are essential in the maintenance of self-renewal and pluripotent capacities of embryonic stem cells (ESCs). It was demonstrated that SALL4 plays an essential role in this process by interacting with two other key regulators in ESCs, Nanog and Oct4. SALL-4 regulates *Oct4* expression by directly binding to the highly conserved regulatory region of *Oct4*, as demonstrated both in vivo and in vitro (De Celis and Barrio, 2009; Wang et al., 2016).

SALL4 (sal-like protein 4) is a newly identified zinc-finger transcription factor and it is one of the SALL gene family (SALL1 to SALL4). It was cloned according to its sequence homology to Drosophila spalt (sal). Alternative splicing generates two variant forms of human SALL4mRNA, SALL4A and SALL4B, and each one of them has a different tissue distribution (Gao et al., 2011).Moreover, it is of particular interest to stem cell biologists because it is linked to the self-renewal of both embryonic stem cells (ESCs) and hematopoietic stem cells (HSCs) also, it is involved in the development of human leukemia (Yang et al.,2012).

Furthermore, some studies found that a novel SALL4/OCT4 transcription regulatory loop is required for balancing the expression of *SALL4* and OCT4 for the maintenance of ESC. While others have observed that a positive feedback relationship is present between *SALL4* and OCT4, the strong self-repression of *SALL4* seems to be the “break” in this loop. In addition, it has been shown that *SALL4* can repress the promoters of other *SALL *family members, such as *SALL1* and *SALL3*, and competes with the activation of these two genes by *OCT4*. Overall these studies suggest that *SALL4* is a master regulator that controls its own expression and the expression of *OCT4*. Moreover, *SALL4* and OCT4 work antagonistically to balance the expressions of other* SALL* gene family members (Yang et al., 2012).

Despite the important shared role of *SALL4* in self-renewal of HSCs and leukemia stem cells (LSCs), some studies have demonstrated that *SALL4* has differential effects on both pro- and anti-apoptotic pathways in normal and leukemic cells (Gao et al., 2011).It was found that during normal hematopoiesis, *SALL4* is preferentially expressed in human *CD34+* hematopoietic stem/progenitors (HS/PCs) and down-regulated in CD34- cells during hematopoietic differentiation (Ma et al., 2006). *SALL4* was aberrantly expressed in many leukemia cell lines and primary leukemia cells of acute myeloid leukemia (AML) and precursor B-cell lymphoblastic leukemia/lymphomas. Moreover, recent studies have shown that*SALL4* gene expression correlates with the disease progression in human chronic myeloid leukemia and its expression in AML patients correlated with treatment status(Lu et al., 2011).

A recent study also revealed that DNA methylation inhibitor 5-azacytidine (5-azaC) caused the demethylation of *SALL4* alleles in NB4 leukemic cells (Yang et al., 2012). The hypomethylation of *SALL4* promoter occurred more frequently in the subtypes of M1 and M2, suggesting that *SALL4* hypomethylation might be associated with the blocked stages of myeloid progenitor cells. Furthermore, *SALL4* hypomethylation was found to be correlated with higher WBCs in AML patients, which may reflect the role of *SALL4* in the control of expansion of leukemic stem cells (Ma et al., 2013).

## Materials and Methods

This study was approved by the ethical committee, review board of National Cancer Institute, Cairo University in accordance with Helsinki guidelines for the protection of human subjects. Our study included 52 adult acute myeloid leukemia (AML) cases. Their age ranged from 19 to 70 years with a median 43 years old. They were 34 females and 18 males. All of them were presented to the medical oncology clinics, National Cancer Institute, Cairo University, during the period of time from December 2014 to June 2015. Ten healthy age and sex-matched subjects were enrolled as control group (collected from donors of Bone Marrow Transplantation). Diagnosis was established after clinical, morphological, cytochemical, flow cytometric and cytogenetic analysis. The diagnosis and classification of AML patients were based on the 2016 World Health Organization (WHO) classification system for tumors of the hematopoietic and lymphoid tissues. After the informed written consent, the samples were collected at presentation, i.e. before receiving any medication. Bone marrow aspirate was the sample of choice for both patients and control. The follow-up data were obtained and the median follow-up duration of these patients was 3 months (range, 3 days– 12 months). Complete remission (CR) was defined according to Dohner et al., 2010 as: Bone marrow blasts <5%; absence of blasts with Auer rods; absence of extramedullary disease; absolute neutrophil count >1.0x 10^9^/L platelet count >100 x 10^9^/L; independence of red cell transfusions.

*Sampling*

Bone Marrow (BM) specimens were collected on potassium ethylene diamine tetra-acetic acid (K-EDTA) (1.5 mg/ mL) for morphologic, immunophenotypic, and molecular analyses, and on sodium heparin for conventional karyotyping and fluorescence in situ hybridization (FISH). Samples are processed within few hours of collection. 

*RNA extraction and cDNA formation*

One ml of bone marrow was collected on EDTA anticoagulant from patients and controls. First, total RNA was extracted from bone marrow cells using QIAamp RNA extraction blood Mini kit (QIAGEN, cat no. 52304) as recommended by the manufacturer’s. The purity and the concentration of the purified RNA was detected using spectrophotometer nano-drop (Quawell, Q-500, Scribner, USA) and stored at – 80^o^C till further assessments.Second, complementary DNA (cDNA) was prepared from RNA using High Capacity cDNA Reverse Transcription Kit (Applied Biosystems, cat no. 4368814) using the four steps thermal cycling conditions (25^o^C for 10 min, 37^o^C for 120 min, 85^o^C for 5 sec then 4^o^C), according to the manufacturer’s instructions. Complementary DNA purity and concentration was detected using spectrophotometer nano-drop (Quawell, Q-500, Scribner, USA) and stored in – 20^o^C till performing quantitative real-time PCR. 

*Real-time RT-PCR*

Real-time quantitative PCR (RQ-PCR) was performed, cDNA was amplified using QuantiTect SYBR Green PCR Master Mix kit (QIAGEN^®^ Austin, Texas, USA catalogue no 204141). The primers of *SALL4* were (Forward: 5’-TGC AGC AGT TGG TGG AGA AC-3’; Reverse: 5’-TCG GTG GCA AAT GAG ACA TTC-3’) (Viviantis, Malais). GAPDH primers were (Forward: 5’-AGA AGG CTG GGG CTC ATT TG-3’ Reverse: 5’- AGG GGC CAT CCA CAG TCT TC-3’) (Viviantis, Malais). Real-time quantitative PCR (RQ-PCR) was performed using 1 μl of cDNA with 9 μl of Master Mix, 0.5 μl of Reverse primer (*SALL4* or *GABDH*), 0.5 μl of Forward primer (*SALL4* or *GABDH*) and 9 μl of Nuclease free H2O with total volume 20 μl. The PCR run was then started on computerized thermocyclers (ABI step one- Applied Biosystems). Real time PCR amplification was performed using PCR program conditions which were 45 cycles of95 °C for 10 min, 95 °C for 15 s,60 °C for 1 s and 72 °C for 30 s. Data from the amplification plot and together with melting curve were obtained and analyzed. To determine the relative expression of *SALL4* gene among the leukemic and normal samples, the comparative threshold cycle (CT) method was used normalizing *SALL4* expression to *GABDH* expression. Here we quantify relative gene expression levels by measuring the CT, an arbitrarily placed threshold which ensures the PCR is in the exponential phase of amplification. The CT is reversely related to the amount of target molecules in the reaction. The classic comparative CT method can be used to calculate the expression level of the gene of interest relative to a calibrator or reference sample using the CT data. 

**Figure 1:**
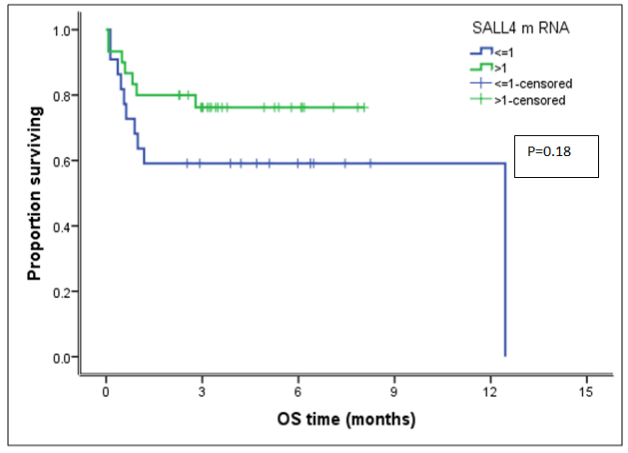
Kaplan-Meier Curve of the Effects of *SALL4* Gene Expression on Overall Survival of AML Patients Included in the Study

**Table 1:** Descriptive Data for Our AML Patients

Variable	No (%)
Sex	
Male	18 (35 %)
Female	34 (65 %)
Age	
Median (range)	43 (19-70)
<50 years	37 (71 %)
≥50years	15 (29 %)
TLCs (x 10^9^/L)	
mean±SD	49.6±87.2
< 50	38/52* (73%)
≥ 50	14/52 (27%)
HB (gm/dL)	
mean±SD	7.8± 1.9
< 8	30/52 (58%)
≥ 8	22/52 (42%)
Platelets (x 10^9^/L)	
mean±SD	77.6± 141.5
< 50	39/52 (75%)
≥ 50	13/52 (25%)
Percent of Peripheral blood blasts	
mean±SD	46.1 %±27.3
< 50	23/52 (44%)
≥ 50	29/52 (56%)
B.M cellularity	
Hypercellular	39/52(75%)
Normocellular	13/52(25%)
Percent of BM blasts	
mean±SD	60.5 %± 23.1
<50%	14/52 (27%)
≥50%	38/52 (73%)
Immunophenotyping markers	
Myeloid	34/52 (65.4%)
Myeloid with CD4 and CD14	11/52 (21%)
Myeloid with aberrant CD7	3/52 (5.8%)
Myeloid with aberrant CD19	2/52 (3.8%)
Myeloid with aberrant CD56	1/52 (1.9%)
Myeloid with CD61	1/52 (1.9%)
CD 34+ve	32/52 (62%)
CD 34 –ve	20/52 (38%)
Cytogenetics	
No mitosis	10/39** (25.6%)
Normal karyotype	9/29*** ( 31.1 % )
t (9; 22)	5/29 (17.2 %)
t (8;21)	3/29 (10.3%)
t (15;17)	4/29(13.8 %)
Complex karyotyping	8/29 (27.6 %)

*52 is the total number of AML cases; **39 is the total number of AML cases undergoing cytogenetic analysis; ***29 is the total number of AML cases undergoing cytogenetic analysis after exclusion of cases showing no mitosis.

**Table 2:** The Expression of *SALL4* m-RNA RQ among the Adult AML Cases and Controls

	*SALL4 mRNA RQ* by RT-PCR			
	Total No	Median	Minimum	Maximum
Cases	52	2	0.1	25
Controls	10	1		
P-value	< 0.001			

**Table 3:** Statistical Comparison of Clinical Data between AML Patients with *SALL4* Gene Expression ≤ 1.0 RQ and Patients >1.0 RQ

	*SALL4* ≤ 1.0 RQ (No=22)	*SALL4* >1.0 RQ (No=30)	Significance P-value
Age (y)	Median:43.5 Range:22-65 Mean±SD: 42±65	Median:42 Range:19-70 Mean±SD: 39.7±14	0.49
Sex			
Female (No= 34)	13 (38.2%)	21 (61.8%)	0.41
Male (No =18)	9 (50.0%)	9(50.0%)	
Hepatomegaly (No= 26)	14 (53.8%)	12 (46.2%)	0.09
Splenomegaly (No= 23)	13 (56.5%)	10 (43.5%)	0.07
Lymphadenopathy (No= 19)	10 (52.6%)	9 (47.4%)	0.25
WBCs(x10^9^/l)	43±62.7	53.9±102.4	0.52
Hb (g/dl)	7.4± 1.4	8.2 ±2.2	0.31
Platelet (x10^9^/l)	45.6±36.5	101.7±181.4	0.47
Peripheral blood blast %	52.3±28.7	41.6±25.7	0.14
BM blasts% at presentation	62.9±22.3	58.7±24.0	0.59

P ≤ 0.05 is considered significant

**Table 4:** Statistical Comparison between the Two *SALL4* Groups According to WHO Classification

*SALL4*		AML with recurrent genetic abnormalities	AML not otherwise specified	Therapy related AML	Total	*P-value*
≤1.0 RQ	Count	3	11	0	14	0.583
	%	10.30%	37.90%	0.00%	48.30%	
>1.0 RQ	Count	4	8	3	15	
	%	13.80%	27.70%	10.3	51.70%	
Total	Count	7	19	3	29	
	%	24.10%	65.60%	10.30%	100%	

**Table 5:** Statistical Comparison between AML Cases with and without t(9;22) Regarding *SALL4* Gene Expression

*SALL4*		AML with t (9;22)	Other cases of AML	Total	P value
≤1.0 RQ	Count	3	14	17	Could not be done because of small numbers in each group
	%	10.30%	48.30%	58.60%	
>1.0 RQ	Count	2	10	12	
	%	6.90%	34.50%	41.40%	
Total	Count	5	24	29	
	%	17.20%	82.80%	100%	

**Table 6:** Cumulative Overall Survival of AML Patients Included in the Study

	Number	Cumulative	Significance
		O.S at 6^th^ month	P- value
*SALL4 *Gene Expression			
≤ 1.0 RQ	22	59.10%	
> 1.0 RQ	30	76.20%	0.18
Whole group	52	68.90%	

*Statistical analysis of the data*

Data management and analysis were performed using Statistical Analysis Systems, SAS vs 8.02. The graphs were done using Harvard Graphics, vs 4.

Numerical data were summarized using means and standard deviations or medians and ranges. Categorical data were summarized as percentages. Mann-Witney test was performed numeric variables. Chi-square test was used to compare groups with respect to categorical data or Fisher’s exact test, for small sample size. To measure the strength of association between two numeric variables Spearman’s correlation coefficient was calculated. A value close to 1 or -1 means a perfect positive or negative correlation, respectively; The closer the value to 1 or -1 you approach, the stronger the association. The overall survival was defined as the time from diagnosis to death or lost to follow-up. The disease free survival was calculated from the time of remission to relapse or death or loss to follow. The overall and disease free survival functions were estimated by the Kaplan and Meier method and compared by the log rank test. All p-values are two-sided. P-values < 0.05 were considered significant.

## Results

*Descriptive data of our AML patients*

The age of the studied cases ranged from 19-70 years with a median of 43 years and mean 40.7±13.5 years. Thirty seven cases aged below 50 years (71%) and 15 cases (29%) were ≥ 50 years. The study involved 18 males (34.6%) and 34 females (65.4%), male to female ratio was 1: 1.9. Hepatomegaly was encountered in twenty six cases out of fifty two 26/52 (50%), while splenomegaly was detected in 23/52 cases (44.2%) and lymphadenopathy was encountered in 19/52 (36.5%)

The distribution of the studied group according to laboratory finding and molecular markers are seen in Table 1.

Conventional cytogenetic study was done to 39 out of 52 cases. No mitosis was detected in 10 cases out of the 39 cases (25.6%). So the analysis was done to the remaining 29 cases out of 52 (55.8%). Nine cases out of 29 (31.1%) had normal karyotype, four cases out of 29 (13.8%) had t (15:17), five cases out of 29 (17.2%) had t (9:22) and three cases out of 29 (10.3%) had t (8;21). While eight cases out of 29 (27.6%) showed complex cytogenetic abnormality which were -2, -3, -7, -11,-13,- 21, -22, +13, +17, +21. 

According to FAB classification, the most commonly encountered FAB subgroup was M2 (25/52; 48.1%) (Five cases out of M2 was relapsed AML), followed by M4 (12/52; 23.1%), M1 (10/52; 19.2%), M3 (4/52; 7.7%), and finally M7 (1/52; 1.9%)

According to WHO classification, we found that 19 out of 29 cases (65.7%) presented with AML not otherwise specified. 7 out of 29 patients (24.0%) was AML with recurrent genetic abnormalities and 3 cases out of 29 (10.3%) presented with therapy related AML. Our 29 cases with successful cytogenetic study were classified according to the 2017 European Leukemia Net (ELN) genetic risk stratification, into 3 risk groups: patients with intermediate risk (Mrozek et al., 2012), they were 9 out of 29 (31.1 %), patients with good risk karyotype, they were 7 out of 29 (24.1%) and patients with poor risk karyotype, they were 13 out of 29 (44.8 %).)

According to response to treatment, we found that complete remission (CR) at D28 occurred in 29 out of the 52 cases (55.8%), while 8 out of the 52 cases had partial response (PR) to induction chemotherapy (15.4%). And 15 out of 52 cases died before day 28 (28.8%).

Kaplan-Meier analysis for our data after a follow up period of 12 months and a median observation time of 3 months with 95 % confidence interval; revealed that the cumulative overall survival at sixth month of all AML patients was (68.9 %). The duration of survival of our patients is ranged from 2 days to 12 months after the date of the diagnosis with a median 3 months.

*SALL4 gene expression in AML patients*

Comparative statistical analysis between cases and controls were done as regard *SALL4* gene expression. The observed data revealed that none of the studied controls expressed *SALL4* > 1.0 RQ and there was a highly statistically significant difference between cases and controls regarding *SALL4* gene expression where all cases showed higher expression of *SALL4* than controls with p value <0.001 as shown in table 2

*Correlation between SALL4 gene expression and clinical characteristics*

Table 3 shows that there was no statistically significant difference in age and sex between the patients with *SALL4* gene expression ≤ 1.0 RQ and patients > 1.0 RQ (p value = 0.49 and 0.41 respectively). Also, there was no statistically significant difference in the clinical data such as hepatomgaly and lymphadenopathy between the patients with *SALL4* gene expression ≤ 1.0 RQ and patients > 1.0 RQ (with p value=0.09 and 0.25 respectively). But, there was a borderline statistically significant difference between cases expressing *SALL4* gene ≤ 1.0 RQ and those > 1.0 RQ as regard splenomegaly (p value=0.07). Where, the *SALL4* gene expression tends to be lower in most cases of splenomegaly and hepatomegaly.

*Correlation between SALL4 gene expression and each of hematological lab findings, FAB classification and the cytogenetic risk*

Statistical comparison of laboratory data between AML patients with *SALL4* gene expression ≤ 1.0 RQ and patients > 1.0 RQ was performed as shown in Table 3. There were insignificant statistical differences between the two *SALL4* subgroups regarding each of WBCs count, Hb level and platelet count with P value: 0.52, 0.31, and 0.47 respectively. But, the statistical correlation could not be done between *SALL4* gene expression and each of FAB classification and the cytogenetic risk because of small number of cases in their subgroups.

*Correlation between SALL4 gene expression and WHO subtypes*

As regard cases with *SALL4* ≤1.0 RQ; we had 3 (21.4%) cases were AML with recurrent genetic abnormalities, 11 (78.6%) were AML not otherwise specified and as regard cases with *SALL4* gene expression > 1.0 RQ, we had 4 (26.7%) were AML with recurrent genetic abnormalities, 8 (53.3%) were AML not otherwise specified and 3 (20%) were therapy related AML. There was no statistical significant difference between the 2 groups as regard their *SALL4* gene expression with p value (0.58) (Table 4)

*Correlation between AML cases with and without t (9;22) regarding SALL4 gene expression*

As regard cases with *SALL4* ≤1.0 RQ we had 3 (10.3%) were with t (9;22) and 14 (48.3%) without t(9;22). As regard cases with *SALL4* gene expression > 1.0 RQ, we had 2 (6.9%) with t (9;22) and 10 (34.5%) without t (9;22). But statistical comparative study according to *SALL4* gene expression could not be done because of small number of cases in each subgroup (Table 5).

*Correlation between SALL4 gene expression and each of the treatment outcome and survival*

As regard cases with *SALL4* ≤1.0 RQ; we had 10 (45.5%) cases were with CR, 3 (13.5%) as were with PR and 9 (41%) were died before day 28. As regard cases with *SALL4* gene expression > 1.0 RQ, we had 19 (63.3%) were with CR, 5 (16.7%) were with PR and 6 (20%) were died before day 28. But statistical comparative study according to *SALL4* gene expression could not be done because of small number of cases in each subgroup. As regard the survival study, the cumulative overall survival (COS) at sixth month of all AML patients was (68.9 %). The COS of AML patients whose *SALL4* gene expression ≤ 1.0 RQ was (59.1%). It is slightely worse than those whose *SALL4* gene expression > 1.0 RQ (76.2%), but there was no statistically significant difference between the two patient groups (P value: 0.18) (Table 6 and Figure 1)

## Discussion

Acute myeloid leukemia (AML) is a genetically heterogeneous clonal disorder characterized by accumulation of acquired genetic alterations in hematopoietic progenitor cells. These alterations disturb normal mechanisms of cell growth, proliferation and differentiation. As a result, there is accumulation of abnormal immature cells in the marrow. These cells which interfere with normal hematopoiesis, can escape into the blood, resulting in signs and symptoms of the disease. Such symptoms include, most prominently, anemia, hemorrhage, infection and their consequences (Estey, 2012). 

Acute leukemias account for approximately 2% of all cancers in the United States, but have a disproportionately large effect on cancer survival statistics. Although leukemia is 10 times more prevalent in adults than in children, it accounts for more than 30% of all childhood cancers. Compared to other types of leukemia, AML is the most deadly (Cherkaoui et al., 2014).

The NCI Cancer Registry presented the cancer incidence rates at national and regional level of Egypt in the period of time between 2008 and 2011 based upon results of National Cancer Registry Program (NCRP). They stratified Egypt into 3 geographical strata: lower, middle, and upper. The incidence of cancer in Egypt was 115/100,000 populations and the incidence of Myeloid leukemia was 1.5% of all newly diagnosed cancer patients (Ibrahim et al., 2014).

*SALL4* is a zinc-finger transcriptional factor and it is a member of the SALL gene family. It is one of the few genes that bridge the self-renewal properties of embryonic stem cells (ESCs), normal hematopoitic stem cells (HSCs) and leukemic stem cells (LSCs) (Gao et al., 2011).

In our study; *SALL4* gene expression showed a highly statistically significant difference between cases and controls with higher expression in cases than controls with p value <0.001. Our findings were consistent with a report for the Institute of hematology, China by Shen et al., 2012, which included 24 newly diagnosed Chinese AML patients and concluded that the *SALL4* expression level was increased in the AML patients (median: 1.051) than healthy individuals (median: 0.394) with p value: 0.009. Also; many other studies are concordant with us; concluded that *SALL4* gene expression was increased in the AML patients than control group; that was done by Chen et al., 2013, in china on 35 AML patients (median *SALL4* 1.43%) and 24 iron deficiency anemic patients as control group (median *SALL4* 0%) (p<0.001), Duan et al., (2013) in Hospital of Dalian Medical University, China, on 60 leukemic patients and 10 normal controls with (p<0.05), which was significantly decreased at complete remission (CR), and Jianghua et al., 2014, China on 50 new acute leukemia cases (median *SALL4* 13.9 in B-ALL, 11.1 in AML) and 15 ITP patients as controls (median *SALL4* 1.0) (p=0.001). Other study that done by Lu and his colleagues, 2011 in Harvard Medical School Boston, USA, that included 23 CML cases have found that the *SALL4* gene was present in 12 CML patients who were in blast crisis but not in 11 CML cases who were in chronic phase. 

In our study, in cases with *SALL4* gene expression >1.0 RQ; there was 1/1 M7 (100%), 16/25 M2 (64%), 7/12 M4 (58%), 5/10 M1 (50%) and, 1/4 M3 (25%). But, the statistical analysis could not be done because of small number of subgroups. In a study done by Chen et al., (2013), who found that *SALL4* gene expression differ significantly with AML FAB subtypes and the frequency of *SALL4* expression was in M2 (86.7%, 13/15) > M3 (75.0%, 6/8) > M1 (60.0%, 3/5) > M4 (14.3%, 1/7), and the difference among the 4 groups was statistically significant (P = 0.008). And a study that was carried out by Wang et al., (2013) at Harvard Medical School, Boston, USA on 55 denovoux MDS, 20 AML on top of MDS, 16 post-treatment MDS patients and 10 healthy donors and evaluated their *SALL4* expression in different WHO subtypes and international prognostic scoring system (IPSS) risk groups. The expression of *SALL4* was significantly higher (p<0.001) in refractory anemia with excess blast type 1 (RAEB-1) (35.06±16.32, n=14) and refractory anemia with excess blast type 2 (RAEB-2) (45.66±16.87, n=13) subtypes when compared with healthy control, but not refractory anemia (RA) (6.62±7.21, n=18) or refractory cytopenia with multilineage dysplasia (RCMD) (8.95±9.72, n=10). These results summarized that *SALL4* gene expression increased in high grade morphology and high IPSS score cases.

In the present study; as regard the re sponse to treatment; the cases with *SALL4* gene expression > 1.0 RQ; showed CR in 19/29 (65.5%) > those with PR 5/8 (62.5%) > those who died before day 28 6/15 (40%), A study done by Shen et al., 2012, who found that the *SALL4* expression level was increased in the AML group (median: 1.051; p=0.009), CML-BC group (median: 1.563; p=0.016) and the different AML subtypes M2 (median: 0.974; p=0.039), M3 (median: 0.799; p=0.083) and M5 (median1.465; p=0.026), it was lower in the M2-CR (median: 0.105; p=0.151) and M3-CR (median: 0.023; p=0.037) groups. Interestingly, the level of *SALL4* expression in the CML-CP (median: 0.093; p=0.213) and CML-CR groups (median: 0.025; p<0.0001) was lower in comparison with the HI group, and the increased *SALL4* expression level in the CML-BC group (median: 1.563) was significantly higher than that in the CML-CP (p=0.001) and CML-CR (p<0.0001) groups. Other study done by Jeong et al., 2011 who showed that AML patients who responded to treatment had decreasing *SALL4* expression throughout the course of treatment, while AML patients with disease relapse or drug resistance had increasing *SALL4* expression, which was correlated with disease progression. Also Duan et al., 2013 showed that *SALL4* mRNA expression significantly decreased at CR in AML and increased slightly higher in relapse than in de novo leukemia group, but the difference was not statistically significant (P > 0.05) and Jianghua et al., 2014, also found, *SALL4* mRNA expression is significantly decreased in complete remission stage (median: 0.98) than in acute phase (median: 28.6) (p<0.001).

In our study, the cumulative overall survival at sixth months of our patients was 68.9 %, and there was no statistically significant difference between the two *SALL4* (≤ 1 RQ (59.1%) and > 1 RQ (76.2%)) groups with (P value: 0.18). This differs than other study done by Wang et al., 2013 in China on 20 AML cases on top of CML, higher level of *SALL4* expression was associated with worse survival rates and *SALL4* level decreased following effective therapy (P<0.05). Our results differ than other study carried out by Ma and his colleagues, 2013 on *SALL4* gene expression in 35 AML patients and revealed a significantly positive correlation between the level of *SALL4* expression and the status of *SALL4 SALL4* hypomethylation (r=0.501, P=0.002); where *SALL4* gene is frequently hypomethylated in AML and hypomethylation was correlated with the high expression of *SALL4* gene. In that study; survival curves for the *SALL4* hypomethylated group and *SALL4* methylated group are done and revealed that the estimated 50% survival time of the *SALL4* hypomethylated group (15%) was worse than that of *SALL4* methylated group (26%), but the difference was not statistically significant (P=0.356).
